# Floquet engineering of tilted and gapped Dirac bandstructure in 1T$^\prime$-MoS$_2$

**DOI:** 10.1038/s41598-022-25898-5

**Published:** 2022-12-09

**Authors:** Andrii Iurov, Liubov Zhemchuzhna, Godfrey Gumbs, Danhong Huang, Wang-Kong Tse, Kathy Blaise, Chinedu Ejiogu

**Affiliations:** 1 Department of Physics and Computer Science, Medgar Evers College of City University of New York, Brooklyn, NY 11225 USA; 2 Department of Physics and Astronomy, Hunter College of the City University of New York, 695 Park Avenue, New York, New York, 10065 USA; 3 Donostia International Physics Center (DIPC), P de Manuel Lardizabal, 4, 20018 San Sebastian, Basque Country Spain; 4 US Air Force Research Laboratory, Space Vehicles Directorate, Kirtland Air Force Base, New Mexico, 87117 USA; 5 Department of Physics and Astronomy, The University of Alabama, Tuscaloosa, AL 35487 USA

**Keywords:** Electronic properties and materials, Quantum optics

## Abstract

We have developed a rigorous theoretical formalism for Floquet engineering, investigating, and subsequently tailoring most crucial electronic properties of 1T$^\prime$-MoS$_2$ by applying an external high-frequency dressing field within the off-resonance regime. It was recently demonstrated that monolayer semiconducting 1T$^\prime$-MoS$_2$ exhibits tunable and gapped spin- and valley-polarized tilted Dirac bands. The electron-photon dressed states depend strongly on the polarization of the applied irradiation and reflect a full complexity of the low-energy Hamiltonian for non-irradiated material. We have calculated and analyzed the properties of the electron dressed states corresponding to linear and circular polarization of a dressing field by focusing on their symmetry, anisotropy, tilting, direct and indirect band gaps. Circularly polarized dressing field is known for transition into a new electronic state with broken time-reversal symmetry and a non-zero Chern number, and therefore, the combination of these topologically non-trivial phases and transitions between them could reveal some truly unique and previously unknown phenomena and applications. We have also computed and discussed the density of states for various types of 1T$^\prime$-MoS$_2$ materials and its modification in the presence of a dressing field.

## Introduction

Floquet theory, or even Floquet engineering, which describes the electronic behavior of a wide range of quantum-mechanical systems under a transient periodic field^1,2^, has become extremely popular over the last several years. It is important to note that recent experimental advances in optical and microwave physics, laser technology and emergent technical applications to condensed-matter quantum optics have enabled the direct experimental verification of such theoretical predictions and their applications as well in actual optoelectronic devices^3–7^.

The effect of an off-resonant dressing field strongly depends on its polarization type and direction. Circularly-polarized dressing field enables opening a band gap and breaking down the time-reversal symmetry in graphene. One important technical challenge addressed by Floquet engineering is confining ballistic electrons within a specific spatial region of an optoelectronic device which is directly related to the Klein paradox^8^ in our early studies on material properties and formation of localized states^9–13^ due to appearance of an induced energy band gap^14,15^. Another example where Floquet engineering plays an important role is provided by the optical control of magnetic properties^16–19^. In contrast, a linearly-polarized optical field leads to an in-plane anisotropy and could affect the sequential-tunneling current of doped electrons for a non-zero polarization angle^20,21^. Additionally, a band gap could also be produced in a high-intensity field regime for circularly-polarized irradiation^22^.

Electron-photon dressed states have been studied in a variety of two-dimensional materials^23,24^, including nanotubes^25–27^, graphene^14,28–30^, silicene^31^, transitional-metal dichalcogenides^32^, dice lattice and $\alpha -\mathcal {T}_3$ materials^33–37^, various types of nanoribbons^38–40^, anisotropic phosphorene^41^ and etc.^42–45^ Particularly, the effect of a circularly-polarized dressing field was examined in silicene, as one of the isotropic limiting cases of 1T$^\prime$-MoS$_2$. Furthermore, it was found that a circularly-polarized field also breaks down the equivalence of two valleys and, most surprisingly, could either increase or decrease the band gap depending on its initial value in stark contrast with any known material.^32^ Meanwhile, it was demonstrated that the off-resonant field could substantially modify transport properties^21,46,47^, RKKY interaction^16,48^, excitonic dynamics, ^49^ and topological signatures of a two-dimensional lattice^34,50–52^. Topological effects on various two-dimensional lattices in the presence of elliptically-polarized light were studied in Ref.^53^. As a popular research topic, there has been a huge effort in investigating the Floquet states in all recently discovered two-dimensional materials, especially, in graphene and topological insulator surface states^54^.

If the initial electronic states of a material areanisotropic (e.g. phosphorene), the anisotropic dispersion will even under an external polarized irradiation. In such a situation, linearly-polarized light would substantially modify the existing anisotropy in the electronic energy dispersion. A left- or right-handed circularly-polarized light could make a difference in the pumped states near the Weyl points and special circular dichroism between their emission spectra^55^. Refs.^56^ and^57^ are devoted to optical control of both structural and electronic properties of Weyl semimetals. Specifically, measuring the photo-current in response to a circularly-polarized mid-infrared light leads to direct determination of a Weyl fermion. Finally, Ref.^58^ explores non-equilibrium transient electronic properties of bulk black phosphorus by using a time-periodic laser field, which gives rise to intriguing topological opto-electroniceffects, e.g. photon-dressed Floquet Dirac semimetal states.

Molybdenum disulfide, MoS$_2$, represents a typical example of a monolayer transition-metal dichalcogenides with a distorted tetragonal crystal structure^59–61^. In general, the monolayer semiconducting MoS$_2$ structure may present a trigonal prismatic coordination of the metal atoms (2H with hexagonal symmetry), as well as octahedral coordination (1T with tetragonal symmetry) or 1T$^\prime$ with a distorted and the most exotic symmetry^62,63^. Here, the latter is exactly the subject of our current study. 1T’-MoS$_2$ has been both theoretically predicted and experimentally synthesized, and becomes a real entity in experiments. Furthermore, the structural modification to molybdenum disulfide was proved to be sufficiently stable, as exemplified by successful direct measurements of quantum spin-Hall effect in this lattice, and even some subtle gate-induced superconductivity^64^.

The 1T$^\prime$-MoS$_2$ is found to be one of the most interesting two-dimensional Dirac materials, which is thermodynamically stable and hence easily synthesized in its semiconducting phase. It was also theoretically predicted to display a strong quantum-spin-Hall effect and great potential for optoelectronic and other applications. Under an external transverse electric field, this material exhibits valley-spin-polarized Dirac bands as well as a phase transition between the topological insulator and a regular band insulator similar to silicene. Its energy band structure also demonstrates a special type of anisotropy which is referred to as tilted Dirac bands^65,66^. This material 1T$^\prime$-MoS$_2$ acquires unique features, such as tunable anisotropy, band gap, spin- and valley-polarized states, and coexistence of different topological phases similarly to silicene^67–71^. Some of these effects can be tuned by the strength of an external electric field, while the rest are modulated by intrinsic spin-orbit coupling gap and other lattice parameters of 1T$^\prime$-MoS$_2$. The zero-gap limit of 1T$^\prime$-MoS$_2$ bands is found in 8-Pmmn borophene which was extensively explored over many years because of an observed strong anisotropy in all of its crucial physical properties^72–76^. Very recently, a plasmonic gain was reported in current-biased tilted Dirac nodes^77^. Their optical conductivity and other properties have also been investigated thoroughly^66,78^. In addition, other monolayer materials with a similar tilted band structure, e.g. TaIrTe$_4$, TaCoTe$_2$ or $\alpha$-SnS$_2$, have been synthesized recently.

The remaining part of this paper is organized as follows. In Sec. 2, we review some essential properties resulting from a low-energy Hamiltonian, dispersion and corresponding electronic states of 1T$^\prime$-MoS$_2$, including tilting and anisotropy as well as direct and indirect band gaps. Section 3 is devoted to deriving and analyzing the electron-photon dressed states under an electromagnetic field with either linear or circular polarization, where we have derived the electron-light interaction Hamiltonian, and then, presented and analyzed the band structure of irradiated dressed states both analytically and numerically. The final conclusions and outlook are discussed in Section 4.

## Model and electronic states in 1T$^\prime$-MoS$_2$

We now present our units and estimate the sought quantities related to a real-world experiment. We define the units of energy and momentum as the Fermi energy in graphene for an experimentally accessible yet quite large electron density $n_e^{(0)} = 10^{11}\,$cm$^{-2}$. This corresponds to the Fermi momentum $k_F^{(0)} = \sqrt{2 \pi n_e^{(0)}} = 7.92 \times 10^7 \,$m$^{-1}$ and the Fermi energy $E_F^{(0)} = \hbar v_F k_F^{(0)} = 8.32\times 10^{-21}\,$J $= 52.02\,$meV. Thus, the unit of length is obtained as $l^{(0)} = 1/k_F^{(0)} = 1.26 \times 10^{-8}\,$m$\backsim 10\,$nm.

Once an external dressing field is applied, electrons in 1T$^\prime$-MoS$_2$ will be affected by irradiation with an intensity $\mathcal {I}_{df} = 10^7 - 10^8 \,$W/m$^2$, which gives rise to the electric field amplitude $E_a = 10^5 \,$V/m from the definition for a beam intensity $\mathcal {I}_{df}= c \, \epsilon _0 \, E_a^2/2$, where $\epsilon _0 = 8.85 \times 10^{-12}\,$m$^{-3}\,$kg$^{-1}\,$s$^4\,$A$^2$ is the vacuum permittivity and $c = 2.99 \times 10^8\,$m/s is the speed of light. Under the off-resonance irradiation condition, $\omega \ge 10^{14} - 10^{15} \,$s$^{-1}$ and photon energy of the dressing field $\hbar \omega \backsim 0.1 - 1 \,$eV so that $\hbar \omega \gg E_F^{(0)}$ can be satisfied. At the same time, these values could still be varied up to an order of magnitude in an accessible experimental arrangement.

The logical starting point for building up our model would be presenting a low-energy Hamiltonian and electronic states for 1T$^\prime$-MoS$_2$. In the vicinity of two inequivalent *K* and $K^{\prime }$ valleys, corresponding to indices $\xi = \pm 1$, the main Hamiltonian

1 $$\begin{aligned} \hat{\mathcal {H}}_{\xi =\pm 1}^{\,1T^{\prime }} ({\varvec{k}}) = V_1 k_x\,{\varvec{\Gamma }}^{(2,0)} + \left[ - \xi V_- {\varvec{\Gamma }}^{(0,0)} - \xi V_+ \, {\varvec{\Gamma }}^{(3,0)} + V_2 \, {\varvec{\Gamma }}^{(1,1)} \right] k_y + \left[ \xi {\varvec{\Gamma }}^{(2,0)} - i r_E \, {\varvec{\Gamma }}^{(2,0)}\cdot {\varvec{\Gamma }}^{(3,0)} \right] \Delta _0 \end{aligned}$$

is linear with respect to wave vector ${\varvec{k}}=\{k_x,k_y\}$, which implies that 1T$^\prime$-MoS$_2$ is a Dirac material. According to Ref.^79^, the components of anisotropic Fermi velocity in Hamiltonian (1) are $V_- = 0.286$, $V_+ = 0.721$, $V_1 = 0.387$ and $V_2 = 0.046$ given in terms of $v_F = 10^{6}\,$m/s for graphene. The spin-orbit coupling gap is $\Delta _0 = 0.81\,E^{(0)}$, $r_E = E_z/E_c$ is the relative value for the our-of-plane electric field, and $E_c$ stands for a critical field at which the band gap in 1T$^\prime$-MoS$_2$ will be closed. It is important to notice that the electronic states and their corresponding energy dispersions in 1T$^\prime$-MoS$_2$ are spin- and valley-polarized, i.e., directly depend on indices *s* and $\xi$.

By following the notations in Ref.^66^, the Hamiltonian in Eq. (1) is written in terms of $4 \times 4$ gamma matrices ${\varvec{\Gamma }}^{(0,0)} = {\varvec{\tau }}_0 \otimes {\varvec{\sigma }}_0$, ${\varvec{\Gamma }}^{(1,1)} = {\varvec{\tau }}_1 \otimes {\varvec{\sigma }}_1$, ${\varvec{\Gamma }}^{(2,0)} = {\varvec{\tau }}_2 \otimes {\varvec{\sigma }}_0$ and ${\varvec{\Gamma }}^{(3,0)} = {\varvec{\tau }}_3 \otimes {\varvec{\sigma }}_0$, where the symbol $\otimes$ represents an outer product (or Kronecker product) and ${\varvec{\tau }}_i$ and ${\varvec{\sigma }}_i$ with $i=0,\,1,\,2,\,3$ are regular $2 \times 2$ Pauli matrices defined in pseudospin and real-spin spaces, correspondingly.

**Figure 1:**
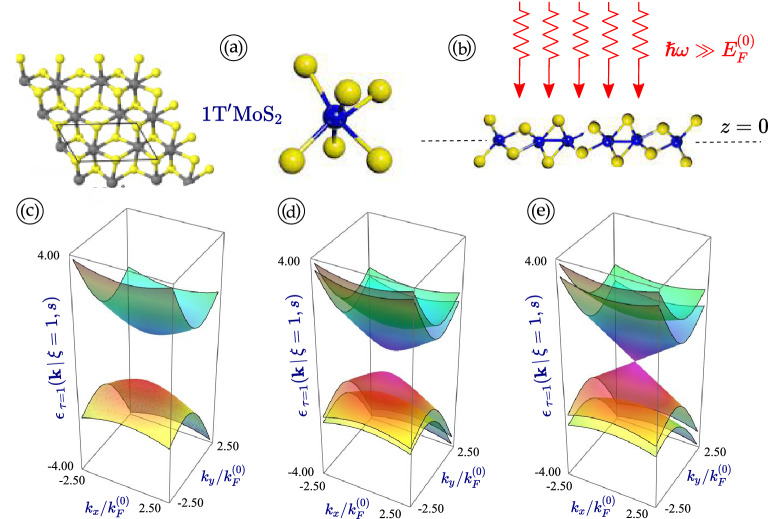
(Color online) Panels (**a**)-(**b**) illustrate a two-dimensional 1T$^\prime$-MoS$_2$ material at $z=0$ in the absence [(**a**)] and presence [(**b**)] of an incident optical field, respectively. On the other hand, panels (**c**)-(*e*) display valley- and spin-polarized energy dispersions $\varepsilon _{\tau =\pm 1}({\varvec{k}} \, \vert \, \xi =1, s)$ for 1T$^\prime$-MoS$_2$ with a finite spin-orbit coupling gap $\Delta _0$ in the absence of optical-dressing field, where three panels correspond to different values of the external electric field $r_E = 0.0$, 0.5, 1.0. Here, we have chosen the valley index $\xi = 1$ for all three cases. Panels (a) - (d) correspond to the different values of the relative electrostatic field $r_E = 0.0$, as labeled.

**Figure 2:**
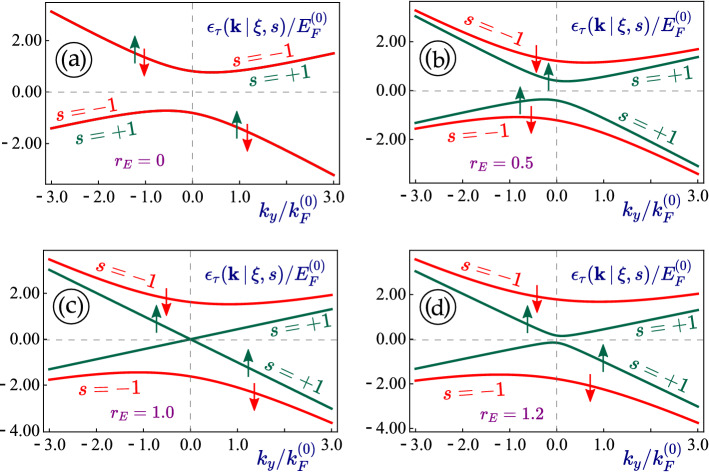
(Color online) $k_y$-dependence of the energy dispersions $\varepsilon _{\tau }(k_x=0, k_y \, \vert \, \xi , s)$ for non-irradiated 1T$^\prime$-MoS$_2$. Each panel corresponds to a different value of the perpendicular electric fields $r_E = 0$, 0.5, 1.0 and 1.2, where the smaller band gap of spin $s=1$ state is closed for $r_E=1$. Here, we take $\xi =1$ and $k_x=0$ for all considered cases.

In a matrix form, we can rewrite the Hamiltonian in Eq. (1) as

2 $$\begin{aligned} \hat{\mathcal {H}}_{\xi =\pm 1}^{\,1T^{\prime }} ({\varvec{k}}) = \left[ \begin{array}{cccc} - (V_+ + V_-)\,\xi k_y &{} 0 &{} r_E \Delta _0 - i V_1 k_x &{} \xi \Delta _0 + V_2 k_y \\ 0 &{} - (V_+ + V_-)\,\xi k_y &{} \xi \Delta _0 + V_2 k_y &{} r_E \Delta _0 - i V_1 k_x \\ 0 &{} 0 &{} (V_+ - V_-)\,\xi k_y &{} 0 \\ 0 &{} 0 &{} 0 &{} (V_+ - V_-)\,\xi k_y \end{array} \right] + H.c. \, , \end{aligned}$$

where *H*.*c*. means a Hermitian conjugate matrix. From Eq. (2), the low-energy dispersions in the vicinity of two Dirac points are immediately obtained as

3 $$\begin{aligned}{} & {} \varepsilon _{\tau }({\varvec{k}} \, \vert \, \xi , s) = - V_-\,\xi k_y + \tau \,\mathbb {S}({\varvec{k}}\, \vert \, \xi , s) \, , \end{aligned}$$

4 $$\begin{aligned}{} & {} \mathbb {S}({\varvec{k}}\, \vert \, \xi , s) = \sqrt{\left[ (\xi - s \, r_E) \Delta _0 + V_2 k_y \right] ^2 +(V_+ k_y)^2 + (V_1 k_x)^2} \, , \end{aligned}$$

where $\tau =\pm 1$ label the electron/hole states related to the conduction and valence bands, while $s=\pm 1$ is the real spin index. It is clear from Eq. (3) that the obtained energy spectrum becomes anisotropic and tilted with respect to $k_y$ axis (i.e., the $-V_-\,\xi k_y$ term gives rise to an equal-magnitude contribution but with opposite sign around $k_y = 0$). Moreover, the energy band gap appears as indirect and closes as $\xi = s\,r_E$. Similarly to silicene, $\xi > s \, r_E$ is a topological insulator phase while $\xi < s \, r_E$ corresponds to a regular band insulator. Only the $r_E$-term in the expression depends on the spin index *s*. Therefore, if we plot the energy dispersions for the case without a perpendicular electric field ($r_E = 0$), there is no spin-splitting in the band structure of electrons, as seen in the corresponding plots of our Figs. 1 and 2.

Corresponding to Eq. (3), the wave functions are given by

5 $$\begin{aligned} \Psi _{\xi =\pm 1}^{\,1T^{\prime }} ({\varvec{k}}) = \left[ \begin{array}{c} s \, \mathcal {D}_{\,s,\,\xi }({\varvec{k}}, \Delta _0) \\ - \mathcal {D}_{\,s,\,\xi }({\varvec{k}}, \Delta _0) \\ 1 \\ -s \end{array} \right] \, \frac{\texttt {e}^{i \xi k_x x} \, \texttt {e}^{i k_y y}}{\sqrt{\mathcal{A}}} \, , \end{aligned}$$

where $\mathcal{A}$ represents the sample area,

6 $$\begin{aligned} \mathcal {D}_{\,s,\,\xi }({\varvec{k}}, \Delta _0) = \left[ \xi V_+ k_y - \tau \, \mathbb {S}({\varvec{k}} \, \vert \, \xi , s) \right] /\left[ (\xi - s r_E) \, \Delta _0 + V_2 k_y - i s V_1 k_x \right] \, \, \end{aligned}$$

and $\mathbb {S}({\varvec{k}} \, \vert \, \xi , s)$ has been given by Eq. (4).

**Figure 3:**
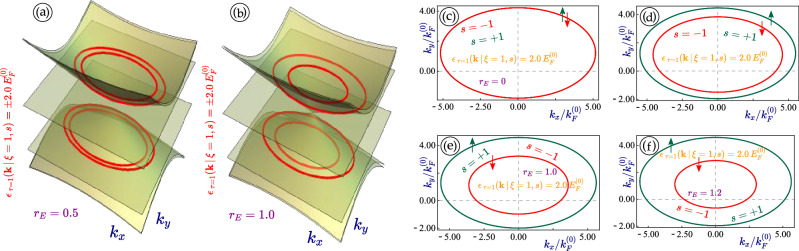
(Color online) Contour plots (**a**)-(**b**) with $r_E =0.5$ and 1.0 and a cut of $\varepsilon _{\tau =\pm 1}(k_x=0, k_y \, \vert \, \xi , s) = 2E^{(0)}$ for non-irradiated 1T$^\prime$-MoS$_2$. For the rest of panels (**c**)-(*f*), each one corresponds to a different value of the perpendicular electric field $r_E = 0$, 0.5, 1.0 and 1.2, where we have chosen $\xi = 1$ for these four plots, respectively.

First, we would like to emphasize that one is facing with highly unusual electronic states even though the Hamiltonian looks linear with respect to two components of the wave vector ${\varvec{k}}=\{k_x,k_y\}$. In this case, although the Fermi velocities $V_+$ and $V_-$ are along the main diagonal of the Hamiltonian matrix, the gap terms, which remain finite at ${\varvec{k}} = 0$, appear as off-diagonal ones. This Hamiltonian structure seems highly unusual and was not encountered in any previously considered materials, e.g. graphene, silicene, regular transition metal dichalcogenides, 8-pmmn borophene or even phosphorene.

The calculated energy dispersions presented in Figs. 1 and 2 are tilted and anisotropic. There is no mirror symmetry with respect to $k_y \leftrightarrow -k_y$, while such symmetry remains for the $k_x$ component of electron momentum, as can be verified from Fig. 3. Generally, there are four non-equivalent Fermi velocities $V_+$, $V_-$, $V_1$ and $V_2$ which affect different properties of 1T$^\prime$-MoS$_2$ and contribute to its anisotropy.

The two subbands in each of the valence and conduction bands rely on the spin index $s=\pm 1$. Thus, we expect two inequivalent subbands and two different band gaps unless no external perpendicular electrostatic field is present or $r_E = 0$. The smaller band gap could be closed at $r_E s = \xi$ and the resulting lattice will reduce to semimetallic. For $r_E < 1$, the band structure represents a topological insulator, while it changes to a regular band insulator if $r_E > 1$. This situation is quite similar to silicene. Additionally, all dispersion curves and band gaps depend on the valley index $\xi = \pm 1$, which makes these electronic states both spin- and valley-polarized.

## Electron dressed states

In this Section, we aim to calculate and analyze new electronic states originating from electron-photon interaction in the presence of an applied high-frequency external optical field. As usual, we refer to these modified electronic states as electron dressed states and an optical field as a dressing field. Quantum mechanically, the vector potential of the dressing field enters into an effective Hamiltonian through a canonical substitution for the electron wave vector ${\varvec{k}}$, which modifies the Hamiltonian in Eq. (1) by using a replacement: $\hbar {\varvec{k}}\longrightarrow \hbar {\varvec{k}}-q{\varvec{A}}(t)$, where *q* is the particle charge and the time-dependent vector potential field ${\varvec{A}}(t)$ can be determined by its polarization and the applied electric-field strength. Meanwhile, the presence of an ${\varvec{A}}(t)$ field will substantially modify the band structure and gaps, as well as the spin-orbit splitting of the material being investigated. In order to solve the associated eigenvalue problem, we have to rely on a perturbation method based on the Floquet-Magnus expansion of an interaction Hamiltonian in powers of inverse frequency, which works particularly well for an off-resonant high-frequency irradiation^80^.

### Linearly-polarized dressing field

In general, ${\varvec{A}}^{(E)}(t)$ for an elliptical (clockwise) polarization can be written as

7 $$\begin{aligned} {\varvec{A}}^{(E)}(t) = \left[ \begin{array}{c} A^{(E)}_x (t) \\ A^{(E)}_y (t) \end{array} \right] = \frac{E_0}{\omega } \left[ \begin{array}{c} \cos \Theta _p \cos (\omega t) - \beta \, \sin \Theta _p \sin (\omega t) \\ \sin \Theta _p \cos (\omega t) + \beta \,\cos \Theta _p \cos (\omega t) \end{array} \right] \, , \end{aligned}$$

where $E_0$ is the electric-field strength, $\Theta _p$ represents the polarization angle of the optical field. Particularly, the vector potential for a linearly-polarized optical field with $\beta = 0$ in Eq. (7) takes the form

8 $$\begin{aligned} {\varvec{A}}^{(L)}(t) = \left[ \begin{array}{c} A^{(L)}_x (t) \\ A^{(L)}_y (t) \end{array} \right] = \frac{E_0}{\omega } \left[ \begin{array}{c} \cos \Theta _p \\ \sin \Theta _p \end{array} \right] \, \cos (\omega t ) \ . \end{aligned}$$

Physically, since the Hamiltonian in Eq. (1) is linear with respect to $k_{x,y}$, under the ${\varvec{A}}^{(L)}(t)$ field it only acquires an additional *interaction* term, yielding

9 $$\begin{aligned} \hat{\mathcal {H}}_{1} ({\varvec{k}}\, \vert \, \tau ) \Longrightarrow \hat{\mathbb {H}}^{(L)}({\varvec{k}}, t) = \hat{\mathcal {H}}_{1} ({\varvec{k}}\, \vert \, \tau ) + \hat{\mathcal {H}}_A^{(L)}(t) \ , \end{aligned}$$

where the ${\varvec{k}}$ independent interaction term is

10 $$\begin{aligned} \nonumber \hat{\mathcal {H}}_A^{(L)}(t)= & {} c_0 \, \cos (\omega t) \\\times & {} \left[ \begin{array}{cccc} \xi \,\left( V_- + V_+ \right) \, \sin \Theta _p &{} 0 &{} i V_1 \cos \Theta _p &{} - V_2 \sin \Theta _p \\ 0 &{} \xi \,\left( V_- + V_+ \right) \, \sin \Theta _p &{} - V_2 \sin \Theta _p &{} i V_1 \cos \Theta _p \\ 0 &{} 0 &{} \xi \,\left( V_- - V_+ \right) \, \sin \Theta _p &{} 0 \\ 0 &{} 0 &{} 0 &{} \xi \,\left( V_- - V_+ \right) \, \sin \Theta _p \end{array} \right] + H.c. \ . \end{aligned}$$

In Eq. (10), we notice an important difference between tilted 1T$^\prime$-MoS$_2$ and all previously studied materials, i.e., the optical-coupling constants $c_0^{(i)} = e V_i E_0/\omega$, corresponding to different Fermi velocities, are not the same for all elements of the Hamiltonian matrix in Eq. (10). The only time-dependent term in Eq. (10) is $\sim \cos (\omega t)$, related to the linearly-polarized irradiation but not to material specification. If the direction of the linear polarization is chosen along the $x-$axis, we have $\Theta _p = 0$ and Eq. (10) is now reduced to

11 $$\begin{aligned} \hat{\mathcal {H}}_A^{(L)}(t) = i \, c_0 V_1 \, \cos (\omega t) \, \left[ \begin{array}{cccc} 0 &{} 0 &{} 1 &{} 0 \\ 0 &{} 0 &{} 0 &{} 1 \\ -1 &{} 0 &{} 0 &{} 0 \\ 0 &{} -1 &{} 0 &{} 0 \end{array} \right] = - \frac{c_0 V_1}{2} \cos (\omega t) \, {\varvec{\Gamma }}^{(2,0)} \, , \end{aligned}$$

where we introduce the notations: $c_0 = v_F e E_0/\omega$ represents the light-electron interaction energy while $\lambda _0 = c_0/(\hbar \omega )$ is the dimensionless light coupling strength.

**Figure 4:**
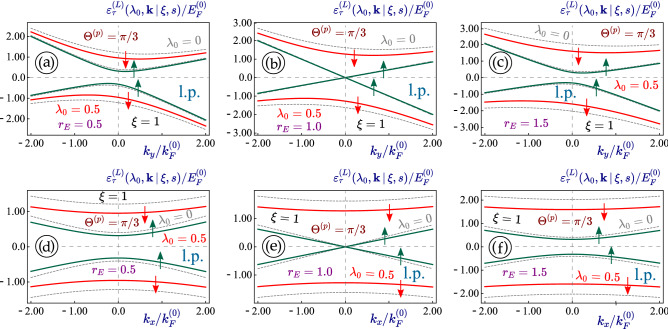
(Color online) Energy dispersions $\varepsilon ^{(L)}_{\tau =\pm 1}(\lambda _0, {\varvec{k}} \, \vert \, \xi = 1, s)$ for the case of linearly-polarized irradiation applied to a 1T$^\prime$-MoS$_2$ lattice. The dispersions for non-irradiated materials $(\lambda _0 = 0)$ and the same lattice parameters are also presented as dashed and gray curves for comparison. Upward and downward spin states with $s=+1$ and $s=-1$ are denoted by green and red arrows, respectively. The linear-polarization angle $\Theta ^{(p)}= \pi /3$ of a dressing field and the electron-photon coupling parameter $\lambda _0 = 0.5$ are chosen for all plots. Three upper panels (**a**)-(**c**) show the initially asymmetric $k_y$-dependence of the electron band structure, while the lower ones (**d**)-(**f**) demonstrate its $k_x$-dependence. Both rows of panels are related to a specific value of external electrostatic field $r_E = 0.5$ [(**a**), (**d**)], 1.0 [semi-metallic; (**b**), (**e**)] and 1.5 [(**c**), (*f*)], as labeled. Here, ${\varvec{K}}$-valley ($\xi =1$) was selected for all plots.

**Figure 5:**
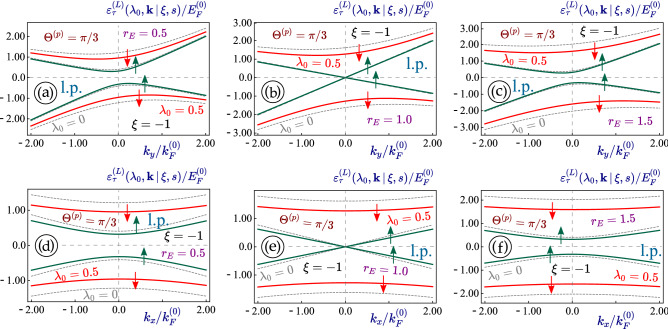
(Color online) Energy dispersions $\varepsilon ^{(L)}_{\tau =\pm 1}(\lambda _0, {\varvec{k}} \, \vert \, \xi = -1, s)$ for the case of linearly polarized irradiation applied to a 1T$^\prime$-MoS$_2$ lattice. The dispersions for non-irradiated materials $(\lambda _0 = 0)$ and the same lattice parameters are also presented as dashed and gray curves for comparison. Upward and downward spin states with $s=+1$ and $s=-1$ are denoted by green and red arrows, respectively. The linear-polarization angle $\Theta ^{(p)}= \pi /3$ of a dressing field and the electron-photon coupling parameter $\lambda _0 = 0.5$ are chosen for all plots. Three upper panels (**a**)-(**c**) show the initially asymmetric $k_y$-dependence of the electron band structure, while the lower ones (**d**)-(**f**) demonstrate its $k_x$-dependence. Both rows of panels are related to a specific value of external electrostatic field $r_E = 0.5$ [(**a**), (**d**)], 1.0 [semi-metallic; (**b**), (**e**)] and 1.5 [(**c**), (*f*)], as labeled. Here, $\xi = - 1$ value of the valley index (${\varvec{K}}^\prime$-valley) was selected for all plots.

In the Floquet-Magnus perturbation approach, which has been widely employed to study the electronic states under an off-resonance and high-frequency dressing field^80,81^, $\hat{\mathcal {H}}_A^{(L)}(t)$ in Eq. (10) can be equivalently written as

12 $$\begin{aligned} \hat{\mathcal {H}}_A^{(L)}(t) = \hat{\mathbb {O}}_{(L)}(c_0 \, \vert \, \xi ) \, \texttt {e}^{i \omega t} + \hat{\mathbb {O}}_{(L)}^{\dagger }(c_0 \, \vert \, \xi ) \, \texttt {e}^{-i \omega t} \ , \end{aligned}$$

in which the time-independent matrix $\hat{\mathbb {O}}_{(L)}(c_0 \, \vert \, \xi )$ has the form

13 $$\begin{aligned} \hat{\mathbb {O}}_{(L)}(c_0 \, \vert \, \xi )= & {} \frac{c_0}{2} \left[ \begin{array}{cccc} (V_+ + V_-)\, \xi \sin \Theta _p &{} 0 &{} i V_1 \cos \Theta _p &{} - V_2 \sin \Theta _p \\ 0 &{} (V_+ + V_-)\, \xi \sin \Theta _p &{} - V_2 \sin \Theta _p &{} i V_1 \cos \Theta _p \\ 0 &{} 0 &{} (V_- - V_+)\, \xi \sin \Theta _p &{} 0 \\ 0 &{} 0 &{} 0 &{} ( V_- - V_+)\, \xi \sin \Theta _p \end{array} \right] + H.c. \nonumber \\= & {} \xi \, c_0 \, \sin \Theta _p \left( V_+ {\varvec{\Gamma }}^{(3,0)} + V_- {\varvec{\Gamma }}^{(0,0)} \right) - c_0 V_1\,\cos \Theta _p \, {\varvec{\Gamma }}^{(2,0)} - c_0 V_2 \sin \Theta _p \, {\varvec{\Gamma }}^{(1,1)} \, . \end{aligned}$$

By using the Floquet-Magnus series expansion up to the powers of $1/\hbar \omega$, the resulting effective Hamiltonian for the dressed state is denoted as $\hat{\mathcal {H}}_{\text {eff}}^{\,(L)}({\varvec{k}}\, \vert \, \tau )$ and calculated by

14 $$\begin{aligned} \hat{\mathcal {H}}_{\text {eff}}^{\,(L)}(\lambda _0, {\varvec{k}}\, \vert \, \xi , r_E)= & {} \hat{\mathcal {H}}_{\xi }^{1T^\prime }({\varvec{k}}\, \vert \, r_E) + \frac{1}{\hbar \omega } \, \left[ \, \hat{\mathbb {O}}_{(L)}(c_0 \, \vert \, \xi ) , \, \hat{\mathbb {O}}_{(L)}^\dagger (c_0 \, \vert \, \xi ) \,\right] \nonumber \\+ & {} \frac{1}{2 (\hbar \omega )^2} \left\{ \left[ \left[ \, \hat{\mathbb {O}}_{(L)}(c_0 \, \vert \, \xi ), \, \hat{\mathcal {H}}_{\xi }^{1T^\prime }({\varvec{k}}\, \vert \, r_E) \, \right] , \, \hat{\mathbb {O}}_{(L)}^{\dagger }(c_0 \, \vert \, \xi ) \, \right] \,\, + \,\, H.c. \right\} \,\, + \cdots \ . \end{aligned}$$

We note that the other $1/\omega ^2$ term proportional to ${1}/{3 (\hbar \omega )^2}$ vanishes for our driving protocol Eq. (10). As expected, the effective Hamiltonian in Eq. (14) can be expressed as the sum of the non-interacting Hamiltonian $\hat{\mathcal {H}}_{\xi =\pm 1}^{1T^\prime }({\varvec{k}}\, \vert \, r_E)$ in Eq. (1) plus some small corrections from the dressing field.

The leading-order correction term $\left[ \, \hat{\mathbb {O}}_{(L)}(c_0 \, \vert \, \xi ) , \, \hat{\mathbb {O}}_{(L)}^\dagger (c_0 \, \vert \, \xi ) \,\right]$ in Eq. (14) is ${\varvec{k}}$-independent and therefore, introduces an irradiation-induced band gap for electrons in dressed states at ${\varvec{k}}=0$. By considering the 1T$^\prime$-MoS$_2$ material as an example, however, the higher-order correction terms can still affect the dispersion relations near ${\varvec{k}} = 0$. For linearly-polarized light, the leading-order correction term in Eq. (14) is always zero since $\hat{\mathbb {O}}_1(c_0 \, \vert \, \xi )$ in Eq. (13) is Hermitian. Additionally, we formally denote the higher-order correction terms in Eq. (14) as $\hat{\mathbb {T}}_2(\lambda _0, {\varvec{k}} \, \vert \, \xi , s)$. Specifically, for $\Theta _p = 0$, the leading contribution of $\hat{\mathbb {T}}_2(\lambda _0, {\varvec{k}} \, \vert \, \xi , s)$ is calculated as

15 $$\begin{aligned} \hat{\mathbb {T}}_2(\lambda _0, {\varvec{k}} \, \vert \, \xi , s)= & {} \lambda _0^2 \, V_1^2 \, \left[ \begin{array}{cccc} \xi \, V_+ \, k_y &{} 0 &{} - r_E \, \Delta _0 &{} - \xi \Delta _0 - V_2 k_y \\ 0 &{} \xi \, V_+ \, k_y &{} - \xi \Delta _0 - V_2 k_y &{} - r_E \, \Delta _0 \\ 0 &{} 0 &{} - V_+\,\xi k_y &{} 0 \\ 0 &{} 0 &{} 0 &{} - \xi \, V_+ \, k_y \end{array} \right] + H.c. = \nonumber \\= & {} (\lambda _0 V_1)^2 \, \left[ \xi \, V_+\, k_y \, {\varvec{\Gamma }}^{(3,0)} - (\xi \Delta _0 + V_2 k_y) \, {\varvec{\Gamma }}^{(1,1)} + r_E \Delta _0 \, {\varvec{\Gamma }}^{(2,0)} \right] \ . \end{aligned}$$

Mathematically, the energy dispersions for a linearly-polarized dressing field could be obtained analytically, but the expression is too long to write it down, even for $\Theta _p=0$. Therefore, we will choose numerical computation of dressed states, instead. It is interesting to note that similarly to graphene, the two gapless branches with $r_E = \xi$ do not acquire any band gap under a linearly-polarized dressing field. The dispersions of such irradiated subbands are

16 $$\begin{aligned} \varepsilon ^{(L)}_{\tau =\pm 1}(\lambda _0, {\varvec{k}} \, \vert \, \xi , s) = -\xi V_-k_y \pm \sqrt{ (V_1 k_x)^2 + \left( 1 - 2 \lambda _0^2 V_1^2 \right) ^2 \, \left( V_2^2 + V_+^2 \right) \, k_y^2} \ , \end{aligned}$$

where $\lambda _0 = v_F e E_0/(\hbar \omega ^2) \ll 1$ and each of the Fermi velocities $V_1$, $V_2$, $V_+$ and $V_-$ is scaled by the Fermi velocity $v_F = 10^6 \,$m/s of graphene. As a comparison, for linearly-polarized dressing field with the same coupling constant $\lambda _0$ applied to graphene and a dice lattice, we get their dispersions $\pm \hbar v_F \,\sqrt{k_x^2 + a(\lambda _0)^2 k_y^2}$, where $a(\lambda _0) = 1 - \lambda _0^2/2$ for graphene whereas $a(\lambda _0) = 1 - \lambda _0^2/4$ for a dice lattice. Linearly-polarized irradiation is generally known to induce or modify the existing anisotropy in electron energy dispersion for two-dimensional lattices. If the initial band gap is zero, it remains zero in either graphene or an $\alpha -\mathcal {T}_3$. Here, we are dealing with an initially anisotropic dispersion, and then, the direction of light polarization becomes critical and the obtained energy dispersions can vary with polarization angles $\Theta _p$ of a linearly-polarized dressing field.

**Figure 6:**
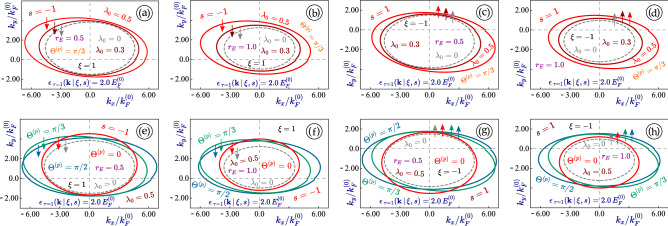
(Color online) Angular dependence of the equienergy $\varepsilon _0^{(L)}({\varvec{k}}) = 2.0 E_F^{(0)}$ cut of the dispersion $\varepsilon _0^{(L)}({\varvec{k}} \, \vert \, \xi , s)$ for 1T$^\prime$-MoS$_2$ in the presence of a linearly-polarized dressing field. The highest subband with $\xi s = - 1$ was selected for all plots. Here, a negative spin index $s= - 1$ (and $\xi = + 1$) was chosen for panels (**a**), (**b**), (**e**), (**f**), while $s= 1$ and $\xi = - 1$ are assumed for plots (**c**), (**d**), (**g**), (**h**). Two different values of the perpendicular electric field $r_E = 0.5$ and $r_E = 1.0$ (semimetallic) were used as labeled. For the upper panels (**a**)-(**d**), each curve corresponds to a different value of the coupling parameter $\lambda _0$, while for the lower ones, it is related to different values of the polarization angle $\Theta ^{(p)}$ as seen. In each plot, a non-irradiated material with $\lambda _0 = 0$ is also shown by a gray dashed curve.

Physically, the assumption for a small irradiation-induced correction to our initial Hamiltonian should be verified first with respect to our calculated energy spectrum. In order to quantify this radiation-field effect, we start with the analytical results in Eqs. (16), (23) and (24) for several limiting cases. Consequently, for chosen values of $E^{(0)}_F$ and $\hbar \omega$, we find that the irradiation-induced corrections to initial bare eigenenergies can be measured by a dimensionless factor $\backsim (c_0/E_F)^2 \backsim 0.05 \, - \, 0.5$ ($c_0$ has an unit of energy) as well as $\backsim \lambda ^2_0 = c^2_0/(\hbar \omega )^2 \sim 0.01 \, - \, 0.1$. Moreover, one should also keep in mind that these two dimensionless parameters, $c_0$ and $\lambda _0$, could be varied independently since $c_0$ is measured with respect to the Fermi energy $E_F$ of the system, while $\lambda _0$ is scaled by the photon energy $\hbar \omega$.

The effect of the linearly polarized dressing field on the energy dispersions in 1T$^\prime$-MoS$_2$ with $\lambda _0 < 1$ is shown in Figs. 4 and 5 for electron states in the *K* and $K^\prime$ valleys with $\xi = \pm 1$, correspondingly. From them, we see that all gaps of the presented energy dispersion relations are reduced as a consequence of the linearly polarized irradiation, i.e., each of the dressed state dispersions shown as a solid red or green line is located at the lower energies compared to the corresponding non-irradiated states. The larger gaps for the electronic states located at higher energies are affected in the most noticeable way so that the subbands with $\xi s = - 1$ demonstrate the largest shift resulting from the applied irradiation.

Importantly, as seen in panels (*b*) and (*e*) of Figs. 4 and 5, the electronic states for $r_E = 1$ do not exhibit any gap and effectively remain gapless under the linearly-polarized light in both $x-$ and $y-$directions in complete analogy to that in graphene or a dice lattice. Since two directions of the energy dispersions are modified at different rates, this leads to a light-induced anisotropy which depends on the polarization angle for the linearly-polarized light. In particular, $k_x$-dispersions acquire a mirror symmetry ($k_x \leftrightarrow -k_x$) even in the presence of a linearly-polarized field. The same feature applies to the $k_y$-subbands’ tilting which remains nearly unaffected by such the dressing field. However, a very interesting observation with different band gaps occurs as one compares two pairs of inner $(\xi s = 1)$ and outer $(\xi s = - 1)$ subbands. In Fig. 4, the outer-pair electron subbands with a larger gap at higher energies are shifted uniformly with little or no dependence on ${\varvec{k}}$, while the inner-pair electron subbands with $(\xi s = 1)$ demonstrates a light-induced modification depending strongly on ${\varvec{k}}$. The appearance looks quite similar to results in Fig. 5 for $\xi =-1$, i.e., with the same amount of asymmetry and gap reduction as those in Fig. 4 at both valleys.

Our numerical computations demonstrate that two pairs of subbands with non-equivalent valley and spin indices respond differently to an applied irradiation, which is expected because of the unique way how these indices enter into the initial Hamiltonian (1) of an irradiated material. What is more surprising is that the linearly-polarized light is also able to affect the bandgap, which is in contrast to all previously investigated Dirac materials.

Since the light-induced anisotropy always appears as the focus in studying effect of linearly-polarized irradiation, it seems very interesting and elucidating to look at the case with nearly elliptical (their actual shape is a lot more complicated than an ellipse except for graphene) constant-energy cuts of the energy dispersions in 1T$^\prime$-MoS$_2$ and explore further how these closed curves are affected by a dressing field.

Our calculated results are presented in Fig. 6, in which we compare the effect of linearly-polarized light with various intensities and polarization directions $\Theta ^{(p)}$ for different valleys of $\xi = \pm 1$. We find from all cases in Fig. 6 that the shift of eccentricity of these ellipses, equivalent to the anisotropy of energy dispersions, increases with enhanced light intensity or the coupling constant $\lambda _0$. Moreover, the orientation of each ellipse clearly depends on the polarization angle $\Theta ^{(p)}$ but not in a monotonic way (e.g. the result at $\Theta ^{(p)} = \pi /2$ closes to that of $\Theta ^{(p)} = 0$ in comparison with $\Theta ^{(p)} = \pi /3$. The irradiation effect looks qualitatively similar for both valleys, however, the initial state, the location of these dispersion ellipses and their tilting are clearly different.

### Circularly-polarized irradiation

The other limit having $\beta = 1$ in Eq. (7) is associated with a circular polarization, i.e.,

17 $$\begin{aligned} {\varvec{A}}^{(C)}(t) = \left[ \begin{array}{c} A^{(C)}_x (t) \\ A^{(C)}_y (t) \end{array} \right] = \frac{E_0}{\omega } \left[ \begin{array}{c} \cos \left( \omega t+\Theta _p \right) \\ \sin \left( \omega t+\Theta _p \right) \end{array} \right] \ . \end{aligned}$$

Since we are seeking stationary and time-independent states, the fixed phase $\Theta _p$ for a circularly-polarized field can be removed without loss of generality. Thus, the vector potential for the circularly-polarized irradiation is simplified to

18 $$\begin{aligned} {\varvec{A}}^{(C)}(t) = \left[ \begin{array}{c} A^{(C)}_x (t) \\ A^{(C)}_y (t) \end{array} \right] = \frac{E_0}{\omega } \left[ \begin{array}{c} \cos \left( \omega t \right) \\ \sin \left( \omega t \right) \end{array} \right] \ . \end{aligned}$$

By employing the vector potential in Eq. (18), the interaction Hamiltonian becomes

19 $$\begin{aligned} \hat{\mathcal {H}}_A^{(C)}(t)= & {} c_0 \left[ \begin{array}{cccc} \xi \, (V_+ + V_-) \, \sin (\omega t) &{} 0 &{} i V_1 \, \cos (\omega t) &{} - V_2 \, \sin (\omega t) \\ 0 &{} \xi \, (V_+ + V_-) \, \sin (\omega t) &{} - V_2 \, \sin (\omega t) &{} i V_1 \, \cos (\omega t) \\ 0 &{} 0 &{} \xi \, (V_- - V_+) \, \sin (\omega t) &{} 0 \\ 0 &{} 0 &{} 0 &{} \xi \, (V_- - V_+) \, \sin (\omega t) \end{array} \right] + H.c.\nonumber \\= & {} c_0 \, \xi \, \left[ (V_- \, {\varvec{\Gamma }}^{(0,0)} + V_+ \, {\varvec{\Gamma }}^{(3,0)}) \, \sin (\omega t) - V_2 \, {\varvec{\Gamma }}^{(1,1)} \, \sin (\omega t) - V_1\, {\varvec{\Gamma }}^{(2,0)} \, \cos (\omega t) \right] \, . \end{aligned}$$

Using the second line in Eq. (19), we can immediately discern the time-independent interaction matrix $\hat{\mathbb {O}}_{(C)}(c_0 \, \vert \, \xi )$ for the case of circularly-polarized light as

20 $$\begin{aligned} \hat{\mathbb {O}}_1(c_0 \, \vert \, \xi )= & {} c_0 \left[ - i \xi \, (V_- \, {\varvec{\Gamma }}^{(0,0)} + V_+ \, {\varvec{\Gamma }}^{(3,0)}) + i V_2 \, {\varvec{\Gamma }}^{(1,1)} - V_1 \, {\varvec{\Gamma }}^{(2,0)} \right] = \nonumber \\= & {} \frac{- ic_0}{2} \, \left[ \begin{array}{cccc} \xi \, (V_+ + V_-) &{} 0 &{} - V_1 &{} - V_2 \\ 0 &{} \xi \, (V_+ + V_-) &{} - V_2 &{} - V_1 \\ V_1 &{} - V_2 &{} \xi \, (V_- - V_+) &{} 0 \\ - V_2 &{} V_1 &{} 0 &{} \xi \, (V_- - V_+) \end{array} \right] \, . \end{aligned}$$

The time-independent effective Hamiltonian for the dressed states is once again obtained by using the Floquet-Magnus expansion, yielding

21 $$\begin{aligned} \hat{\mathcal {H}}_{\text {eff}}^{\,(C)}(\lambda _0, {\varvec{k}}\, \vert \, \xi , r_E)= & {} \hat{\mathcal {H}}_{\xi }^{1T^\prime }({\varvec{k}}\, \vert \, r_E) + \frac{1}{\hbar \omega } \, \left[ \, \hat{\mathbb {O}}_{(C)}(c_0 \, \vert \, \xi ) , \, \hat{\mathbb {O}}_{(C)}^\dagger (c_0 \, \vert \, \xi ) \,\right] \nonumber \\+ & {} \frac{1}{2 (\hbar \omega )^2} \left\{ \left[ \left[ \, \hat{\mathbb {O}}_{(C)}(c_0 \, \vert \, \xi ), \, \hat{\mathcal {H}}_{\xi }^{1T^\prime }({\varvec{k}}\, \vert \, r_E) \, \right] , \, \hat{\mathbb {O}}_{(C)}^{\dagger }(c_0 \, \vert \, \xi ) \, \right] \,\, + \,\, H.c. \right\} \,\, + \cdots \ , \end{aligned}$$

which looks similar to Eq. (14). From Eq. (20), we easily find the first-order correction in the series expansion as

22 $$\begin{aligned} \frac{1}{\hbar \omega } \, \left[ \, \hat{\mathbb {O}}_{(C)}(c_0 \, \vert \, \xi ) , \, \hat{\mathbb {O}}_{(C)}^{\dagger }(c_0 \, \vert \, \xi ) \,\right] = \frac{c_0^2 \, V_1}{\hbar \omega } \, \left[ \begin{array}{cccc} 0 &{} V_2 &{} V_+\,\xi &{} 0 \\ V_2 &{} 0 &{} 0 &{} V_+\,\xi \\ V_+\,\xi &{} 0 &{} 0 &{} -V_2 \\ 0 &{} V_+\,\xi &{} - V_2 &{} 0 \end{array} \right] \, . \end{aligned}$$

The corresponding energy dispersions of the dressed states are given by

23 $$\begin{aligned} \varepsilon _{\tau =\pm 1}({\varvec{k}} \, \vert \, \xi , s) = -V_- \,\xi k_y \pm \sqrt{\widetilde{\Delta }^2_{\xi \, \vert \, s}(\lambda _0) + \left[ \, (\xi - s \, r_E) \Delta _0 + V_2 k_y \right] ^2 + (V_+ k_y)^2 + (V_1 k_x)^2} \ , \end{aligned}$$

where the additional irradiation-induced band gap $\widetilde{\Delta }_{\lambda _0}(\xi \, \vert \, s)$ takes the explicit form

24 $$\begin{aligned} \widetilde{\Delta }_{\xi \, \vert \, s}(\lambda _0) = \pm \sqrt{2 c_0^2 V_1 V_+ \Delta _0 \,(1 - s \, \xi \, r_E) + c_0^4 V_1^2 (V_2^2 + V_+^2)\,} \ , \end{aligned}$$

and it always leads to an enhanced direct bandgap at ${\varvec{k}}= 0$ from Eq. (23). Here, the finite-${\varvec{k}}$ terms in Eq. (23) for dispersions are clearly not affected by the presence of a circularly-polarized irradiation since only the first-order correction will be taken into account.

The first-order $\backsim 1/(\hbar \omega )$ term of the expansion in Eq. (14) is on the order of $c_0^2/(\hbar \omega ) = c_0 \lambda _0$, while the second-order term is determined as $\backsim \lambda _0^2$. These two coefficients are related quantitatively as $E_F^{(0)}/(\hbar \omega )$, which depends on the electron density in a specific material and the range of photon energies. For the analytical result in Eq. (23), $\hbar \omega \gg E_F^{(0)}$ and $\lambda _0 \ll c_0/\hbar \omega \ll 1$ so that the second-order correction to the expansion in Eq. (14) could be safely neglected. In general, however, the situation could change, depending on doping density of the electrons ans well as the irradiation-frequency regime. In fact, if $c_0/\hbar \omega \backsim \lambda _0$, the second-order expansion term will have a considerable effect on the direct band gap, and furthermore, the circularly-polarized field gives rise to a gap reduction if the initial gap is large enough.

**Figure 7:**
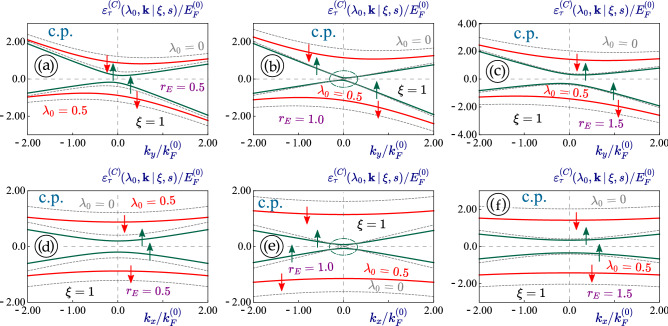
(Color online) Energy dispersions $\varepsilon ^{(C)}_{\tau =\pm 1}(\lambda _0, {\varvec{k}} \, \vert \, \xi = 1, s)$ for the case of a circularly-polarized irradiation applied to a 1T$^\prime$-MoS$_2$ lattice. The dispersions for non-irradiated materials $(\lambda _0 = 0)$ and the same lattice parameters are also shown as dashed and gray curves for comparison. Upward and downward spin states with $s=+1$ and $s=-1$ are denoted by green and red arrows, respectively. Panels (*a*)-(**c**) present the initial asymmetric $k_y$-dependence of electron band structure, the lower panels (**d**)-(**f**), on the other hand, demonstrate their $k_x$-dependence. As labeled, each two panels are related to each other by a selected value for external electrostatic field $r_E = 0.5$ [(**a**), (**d**)], 1.0 [semi-metallic; (**b**), (**e**)] and 1.5 [(**c**), (*f*)]. Here, ${\varvec{K}}$-valley with $\xi =+1$ was considered for all plots.

**Figure 8:**
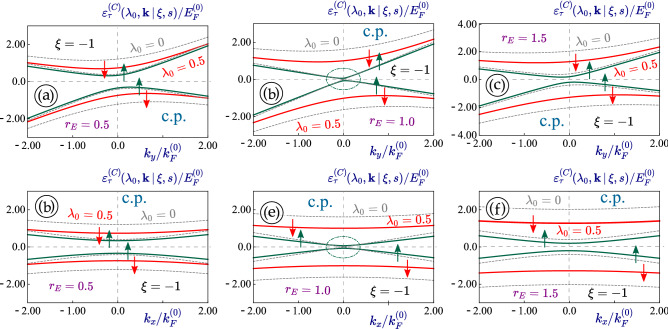
(Color online) Energy dispersions $\varepsilon ^{(C)}_{\tau =\pm 1}(\lambda _0, {\varvec{k}} \, \vert \, \xi = -1, s)$ for the case of a circularly-polarized irradiation applied to a 1T$^\prime$-MoS$_2$ lattice. The dispersions for non-irradiated materials $(\lambda _0 = 0)$ and the same lattice parameters are also presented as dashed and gray curves for comparison. Upward and downward spin states with $s=+1$ and $s=-1$ are indicated by green and red arrows, respectively. Panels (**a**)-(**c**) show the initially asymmetric $k_y$-dependence of electron band structure, while the lower panels (**d**)-(**f**) demonstrate their $k_x$-dependence. As seen, each two panels are connected to each other by a selected value for external electrostatic field $r_E = 0.5$ [(**a**), (**d**)], 1.0 [semi-metallic; (**b**), (**e**)] and 1.5 [(**c**) and (*f*)]. Here, ${\varvec{K}}^\prime$-valley with $\xi = - 1$ was selected for all plots.

**Figure 9:**
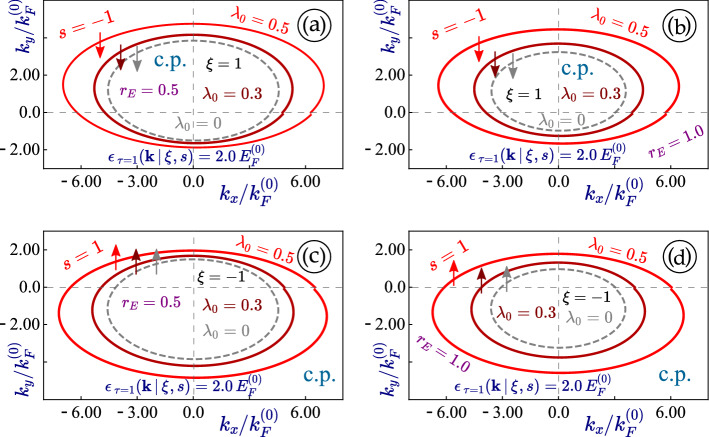
(Color online) Angular dependence of the constant-energy $\varepsilon _0^{(L)}({\varvec{k}}) = 2.0 E_F^{(0)}$ cut of the dispersions $\varepsilon _0^{(C)}({\varvec{k}}\, \vert \, \xi , s)$ for 1T$^\prime$-MoS$_2$ under linearly-polarized dressing field. The highest subband with $\xi \, s = - 1$ was considered in all plots. A negative spin index $s= - 1$ (and $\xi = + 1$) was chosen for panels (**a**), (**b**), while $s= 1$ and $\xi = - 1$ are selected for plots (**c**), (**d**). Two different values for the perpendicular electric field $r_E = 0.5$ [(*a*), (*c*)] and $r_E = 1.0$ [semi-metallic; (**a**), (**c**)] were assumed as labeled. In each plot, a non-irradiated material with $\lambda _0 = 0$ is represented by a dashed gray curve.

**Figure 10:**
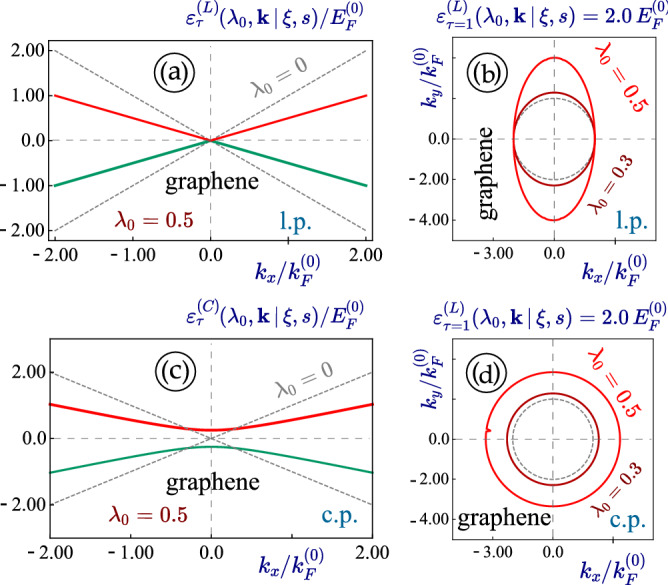
(Color online) Energy dispersions for irradiated *graphene*-like simplification of 1T$^\prime$-MoS$_2$ Hamiltonian in Eq. (1). In particular, we set $V_+ = V_- = 0$, $\Delta _0 = 0$ and $V_1 = V_2 = v_F$, which reduces to regular graphene energy spectrum in Eq. (3). Panels (**a**), (**b**) demonstrate the effect of a linearly-polarized dressing field on such a material, while panels (**c**), (**d**) are associated with a circularly-polarized irradiation. In contrast to familiar dispersion plots $\varepsilon _\tau (\lambda _0,k)$ [in (**a**), (**c**)], panels (**b**), (**d**) present a constant-energy cut [$\varepsilon _\tau (\lambda _0,k) = 2.0 \,E_F^{(0)}$] of two degenerate electron-like energy subbands corresponding to $\tau = 1$.

**Figure 11:**
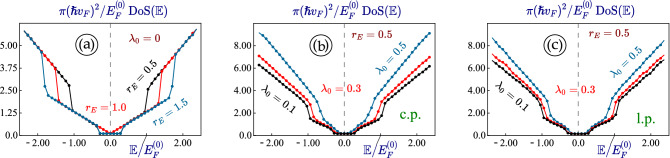
(Color online) Density of states Dos$(\mathbb {E})$ of 1T$^\prime$-MoS$_2$ under an optical-dressing field. Panel (**a**) demonstrates $\text {DoS}(\mathbb {E})$ for non-irradiated 1T$^\prime$-MoS$_2$ layer with different values of electrostatic-field strength: $r_E = 0.5$ (topological insulator phase), $r_E = 1.0$ (spin- and valley-polarized semimetal) and $r_E = 1.0$ (normal band insulator). Plots (**b**), (**c**) represent Dos$(\mathbb {E})$ of 1T$^\prime$-MoS$_2$ with $r_E = 0.5$ and a finite energy gap under both circularly- and linearly-polarized dressing field, separately. For both (**b**) and (**c**), each curve corresponds to a different value of light-electron coupling constant $\lambda _0$ as labeled.

The energy dispersion relations of 1T$^\prime$-MoS$_2$ in the presence of circularly-polarized light are presented in Figs. 7-8 for $c_0/\hbar \omega \backsim \lambda _0 < 1$. Here, we pay special attention to the modification of energy band gap since circularly-polarized light in graphene and $\alpha -\mathcal {T}_3$ materials is known to open a finite energy band gap. However, what we have found is that, for ${\varvec{k}}=0$, an existing direct band gap could be either increased or decreased depending on its initial value: i.e. larger gaps tend to be reduced by a circularly-polarized dressing field. This situation is similar to silicene and expected for 1T$^\prime$-MoS$_2$ since two materials become identical at ${\varvec{k}}=0$. It is also important to note that for circularly-polarized light, the time- and ${\varvec{k}}$-independent matrix $\hat{\mathbb {O}}^{(P)}(c_0 \,\vert \, \xi )$ is not Hermitian, directly leading to opening a noticeable gap ( $\backsim \hbar \omega$) for all known Dirac materials.

For the metallic cases with $r_E=1$, a band gap is still open but smaller than that in graphene. Here, larger band gaps are reduced, which holds true for both inner and outer subdands. The $k_x \leftrightarrow -k_x$ symmetry is kept unchanged under circularly-polarized light. Meanwhile, we expect that the second and third terms in Eq. (21) are proportional to $\sim c_0 \lambda _0$ and $\sim \lambda _0^2$, respectively, and they present opposite roles with respect to gap modification and compete with each other. Similar situation occurs for linearly-polarized light, but the circularly-polarized dressing field changes the outer subbands $(\xi s = - 1)$ stronger and uniformly for all wave vectors ${\varvec{k}}$, in contrast to the inner low-energy subbands.

The orientation and anisotropy for the constant-energy cut of dispersions in 1T$^\prime$-MoS$_2$ remains unchanged under circularly-polarized irradiation, as verified by Fig. 9. These results are quite unique compared to the case with a linearly-polarized dressing field. From figures 4 - 9, we find that the modifications to the eigenenergies by an irradiation field depend on their initial values under no irradiation and usually fall within the $0.1 \,-\,0.3$ range.

The effect of dressing field on graphene has been known for a while now. Therefore, checking this limiting case can be viewed as a starting test for our computation technique rather than an attempt to extract any new information. In fact, for graphene, we can simply set $V_- = V_+ = 0$, $\Delta _0 = 0$ and $V_1 = V_2 = v_F$ for the Hamiltonian in Eq. (1). By doing so, we are left with two off-diagonal blocks relevant to graphene except that two matrix rows are switched. The resulting isotropic energy dispersions $\varepsilon _\tau (k) = \tau \, \hbar v_F k$ are the same as those for graphene, which could also be obtained from Eq. (3) by employing the above simplifications. The comparison of irradiation with different polarizations in such a lattice is shown in Fig. 10, from which we easily find that linearly-polarized light will introduce a substantial anisotropy in energy dispersion while circularly-polarized field tends to open a finite energy band gap.

### Density of states

It is interesting to calculate and analyze the density of states Dos$(\mathbb {E})$ for various phases of 1T$^\prime$-MoS$_2$ materials with the band gaps determined by the dressing-field strength $r_E$ for various types of polarization. The Dos$(\mathbb {E})$ is generally calculated through

25 $$\begin{aligned} \text {DoS}(\mathbb {E}) = \sum \limits _{\tau = \pm 1} \, \sum \limits _{\xi ,s = \pm 1} \, \int \frac{d^2{\varvec{k}}}{(2 \pi )^2} \, \delta \left[ \mathbb {E} - \varepsilon _{\tau =\pm 1}^{(P)}({\varvec{k}} \, \vert \, \xi , s) \right] \, , \end{aligned}$$

where $\delta (x)$ is the Dirac delta function and $\varepsilon _{\tau =\pm 1}^{(P)}({\varvec{k}} \, \vert \, \xi , s)$ are the energy dispersions in the existence of a dressing field with polarization (*P*) (linear, circular or, in general, elliptical). We also note that all energy subbands, including valley, spin and electron-hole index, contribute to Dos$(\mathbb {E})$.

The calculated results for DoS$(\mathbb {E})$ are displayed in Fig. 11, which highlight the band structure of electronic states in 1T$^\prime$-MoS$_2$. DoS$(\mathbb {E})$ is definitely zero within the gap region for each of subbands so that the gap edges appear as jumps (discontinuities) in DoS$(\mathbb {E})$.

For all Dirac materials, DoS$(\mathbb {E})$ becomes an important indicator for the change of Fermi velocity (the group velocity of its dispersion) since gap opening $\hbar v_F k \, \longrightarrow \, \sqrt{ (\hbar v_F k)^2 + \Delta ^2}$ has no effect on DoS$(\mathbb {E})$ outside the gap region, as long as $v_F$ remains unchanged. Meanwhile, DoS$(\mathbb {E})$ shows a strong dependence on the Fermi velocity $\backsim 1/v_F^2$, implying that growth of the DoS$(\mathbb {E})$ under a dressing field originates from a reduction of $v_F$. Such a feature is not seen directly from the energy band structure of irradiated 1T$^\prime$-MoS$_2$. Instead, one finds a substantially enhanced DoS$(\mathbb {E})$ beyond the gap energy for both types of dressing-field polarization. Moreover, this DoS$(\mathbb {E})$ enhancement appears a lot more visible for a circularly-polarized irradiation.

## Summary and remarks

The current work is devoted to an effort for exploring new types of low-energy dispersion and electronic states in recently discovered 1T$^\prime$-MoS$_2$. Such a study is accomplished by utilizing the so-called Floquet engineering, which involves modeling certain technologically intriguing properties of unique electronic states within a two-dimensional lattice after applying a non-resonant high-frequency optical-dressing field. This represents one of the most promising and active research directions in materials science and quantum optics in present days. From a historical perspective, this theoretical approach was originally invented as an effective technique for investigating time-dependent differential equations with periodic coefficients. ^82^

There exists a fundamental research interest in this topic, which connects various aspects of condensed-matter physics and low-dimensional material science with laser optics. This stems from the fact that one can reveal some exceptional, unusual and technologically promising electronic properties of a novel material even before it is actually fabricated and tested experimentally. The resulting electron dressed state physically represents a quasi-particle which combines the essential features of both an irradiated material and an incident optical dressing field. The properties of these produced dressed states can be altered drastically by selecting a specific polarization for an applied irradiation.

In the present work, we have performed a rigorous theoretical and numerical investigation into the dressed electronic states in 1T$^\prime$-MoS$_2$ under an external radiation with either linear or circular polarization. We have derived a number of closed-form explicit expressions for band structure of electrons driven by an external irradiation in the terahertz regime as several specific limiting cases. Meanwhile, we have further demonstrated that analytical results for all four different energy subbands can be generally obtained but the resulting expressions are just too long to write them down.

We have also revealed that linearly- and circularly-polarized light have some similar effects on 1T$^\prime$-MoS$_2$, which have not been predicted in any previous work on Dirac materials. For example, linearly-polarized light, widely known to induce or modify in-plane anisotropy in energy dispersion of a two-dimensional lattice, would also change substantially the band gap in this system. Meanwhile, applying a circularly-polarized irradiation primarily leads to a renormalization of existing band gaps.

One of the most important findings in this research is that not only the initial states of 1T$^\prime$-MoS$_2$ but also their modification by the dressing field depends sensitively on the valley index $\xi = \pm 1$. This includes the switching to direct or indirect band gap, band-structure tilting and the change of Fermi velocities along different wave-vector directions. Consequently, our calculated dressed states present important developments for currently promising valleytronic applications.

All our discovered features of dressed band structure can be further understood through computing density of states of electrons for irradiated 1T$^\prime$-MoS$_2$. The electron density of states represents a strong independent interest to researchers in this field since it is directly related to a handful of crucial collective electronic properties of a material and could be directly measured at the same time.

From the perspective of modern two-dimensional-material based electronics, 1T$^\prime$-MoS$_2$ represents one of the most important player among other nano-materials and a novel representative of an already famous family of transition metal dichalcogenides. The theoretical results in this work on modified electron dispersions could be verified directly by using transmission electron microscopy, scanning tunneling spectroscopy, angle-resolved photo-emission spectroscopy. We are also confident that this work will make a significant contribution to an unprecedented research effort on Floquet engineering among all innovative two-dimensional Dirac materials and other low-dimensional structures.

## Data Availability

All data generated or analyzed during this study are included in this published article.
